# Effects of biologic therapy on novel indices of lung inhomogeneity in patients with severe type-2 high asthma

**DOI:** 10.1136/bmjresp-2024-002721

**Published:** 2025-02-08

**Authors:** Asma Alamoudi, Lorenzo Petralia, Nicholas M J Smith, Haopeng Xu, Dominic Sandhu, Graham Richmond, Nick P Talbot, Grant AD Ritchie, Ian Pavord, Peter A Robbins, Nayia Petousi

**Affiliations:** 1 Department of Physiology, Anatomy and Genetics, University of Oxford, Oxford, UK; 2 Department of Respiratory Care, Prince Sultan Military College of Health Sciences, Dhahran, Saudi Arabia; 3 Department of Chemistry, Physical and Theoretical Chemistry Laboratory, University of Oxford, Oxford, UK; 4 University of Innsbruck Breath Research Institute, Innsbruck, Austria; 5 Nuffield Department of Medicine, University of Oxford, Oxford, UK; 6 Oxford Centre for Respiratory Medicine, Oxford University Hospitals NHS Foundation Trust, Oxford, UK

**Keywords:** Asthma, Respiratory Measurement, Lung Physiology

## Abstract

**ABSTRACT:**

**Introduction/Aim:**

Lung inhomogeneity measures obtained using computed cardiopulmonography (CCP) are sensitive to small-airways disease. Here, we assessed changes in lung inhomogeneity in patients with type-2 high asthma treated with biological therapy and explored the relationship between inhomogeneity measures and conventional asthma disease markers.

**Methods:**

This was an observational study of 91 severe type-2 high asthma patients recruited from a tertiary asthma clinic, of whom 67 subsequently started anti-IL5 or anti-IL5R biologics. Patients were evaluated at baseline and, 54 of those commencing biologics, at their fourth injection with either mepolizumab or benralizumab. Assessments included prebronchodilator and postbronchodilator CCP and spirometry, and measurements of blood eosinophil count (BEC), fractional exhaled nitric oxide and Asthma-Symptom Questionnaire (ACQ-5).

**Results:**

Bronchodilation significantly reduced σlnCl, a novel CCP-derived ventilation inhomogeneity index, (ΔσlnCl −0.08, 95% CI (−0.10 to –0.05), p<0.001). Baseline σlnCl, but not forced expiratory volume in 1 s (FEV_1_) % predicted, was significantly associated with BEC (linear mixed-effects (LME) regression coefficient for BEC 0.18, 95% CI (0.04, 0.32), p=0.01). Following biologics, improvements in σlnCl were significantly dependent on BEC (LME regression coefficient +0.19, 95% CI (0.11, 0.27), p<0.001) whereas improvements in FEV_1_ % predicted related to both BEC and ACQ-5 responses (LME coefficients: BEC −10.8 % pred, 95% CI (−16.1,–5.5); ACQ-5 –3.5 % pred, 95% CI (−5.1 to –1.9), p<0.001). Following biologics, the change in σlnCl followed a bimodal distribution that dichotomised patients into σlnCl-Responders and σlnCl-Non-Responders. Responders, unlike Non-Responders, experienced significant improvements in symptoms and FEV_1_ % predicted (Δ pre-BD FEV_1_15±15% pred, p<0.001) and included a higher proportion of patients in clinical remission at 1 year.

**Conclusion:**

σlnCl is strongly associated with systemic eosinophilic inflammation in severe type-2 high asthma. An early σlnCl response following anti-IL5 biologics identifies patients more likely to experience improvements in symptoms and lung function when systemic eosinophils are depleted. σlnCl may provide a sensitive route for tracking inflammation involving the small airways.

**WHAT IS ALREADY KNOWN ON THIS TOPIC**

- Computed cardiopulmonography (CCP) is a new technique that quantifies inhomogeneity (unevenness) in ventilation and gas exchange, and previous studies showed that σlnCl, a novel CCP-derived index of ventilation inhomogeneity, is very sensitive to early small-airway changes.

**WHAT THIS STUDY ADDS**

- This study shows that σlnCl is strongly associated with the level of systemic eosinophilic inflammation in patients with severe type-2 high asthma. Following biologics, those patients with an early (3–4 months) improvement in σlnCl are likely to experience significant improvements in symptoms and lung function.

**HOW THIS STUDY MIGHT AFFECT RESEARCH, PRACTICE OR POLICY**

- σlnCl may provide an early treatment response signal for improvements in disease activity within (the small airways of) the lung. Future research should use inhomogeneity and other small-airway indices in trials evaluating earlier introduction of biologics in milder disease.

## Introduction

 Asthma affects more than 300 million people worldwide, causing significant morbidity and mortality.^1^ Currently we rely on spirometry as the standard lung function test for diagnosis and monitoring in asthma. In asthma, there is a recognised discordance between lung function measures and clinical markers of disease burden or disease control, including symptoms, inflammatory biomarkers, exacerbation frequency and response to treatment.^2 3^ The introduction of targeted biological therapy has revolutionised the management of severe asthma, with improvements in quality of life, reduction in oral corticosteroid use and decrease in frequency of exacerbations.^4^,^6^ However, the effects of anti-IL5/anti-IL5R monoclonal antibodies — the main biologics in clinical use at the time of our study — on lung function have been less clear.

There have been increasing efforts over the years to develop new techniques for assessing disease activity and lung function. Our research group has recently developed an innovative technique, termed computed cardiopulmonography (CCP), for the assessment of lung function through measurements of inhomogeneity (unevenness) in gas-exchange.^7 8^ The test is non-invasive, non-aerosol-generating, simple to perform and well tolerated in patients with respiratory disease. It provides a multidimensional assessment of lung physiology via novel lung function indices.^7 8^

In recent studies using this technique, we showed that the CCP-derived lung function indices are highly abnormal even in mild COPD and change according to COPD severity.^9^ In patients who have had COVID-19, depending on severity, we found increased anatomical deadspace, reduced absolute lung volumes and increased ventilation inhomogeneity, changes missed using conventional lung function measures.^7^ Additionally, σlnCl, a specific CCP-derived inhomogeneity index that reflects ventilation inhomogeneity, proved a sensitive marker for early airways-related abnormalities: it discriminated young patients with cystic fibrosis and normal forced expiratory volume in 1 s (FEV_1_) from healthy controls,^10^ and young people with modest smoking history (>5 pack-year) but with no respiratory disease from healthy controls.^10 11^ Our first study in asthma found that σlnCl was significantly higher in patients compared with healthy controls; moreover, it correlated better with disease control than FEV_1_, predicting those patients where treatment escalation was clinically required, suggesting that it might be a promising sensitive marker of small-airway change.^12^

Following our initial results with CCP, in this study, we focused on patients with severe type-2 high asthma, the target population for anti-IL5/anti-IL5R monoclonal antibodies, to explore (1) the relationship of these novel inhomogeneity indices with other parameters and characteristics in this patient cohort, such as inflammatory biomarkers and symptoms and (2) whether the novel CCP inhomogeneity indices change with therapy.

## Methods

### Patient and public involvement

None

### Study participants and protocol

Participants with asthma were recruited from the Special Airways Clinic at Oxford University Hospitals. At this clinic, patients are phenotyped using biomarkers—fractional exhaled nitric oxide (FeNO) and blood eosinophil count (BEC)—and categorised as T2-high asthma (when FeNO and/or BEC are elevated) or T2-low asthma. The study was structured alongside the clinical care pathway and included only patients with severe T2-high asthma (as defined by GINA (Global Initiative for Asthma) and on GINA treatment steps 4 or 5, but not on biologic therapy at the time of recruitment to the study).^13^ Recruitment began in June 2018 but was halted because of the COVID-19 pandemic in February 2020. Recruitment restarted in November 2020 with the last patient completing in September 2023. This research was approved by the South Central–Oxford A Research Ethics Committee (Ref: 17/SC/0172) and registered at ISCRNT (Ref: 12223638).

**Figure 1:**
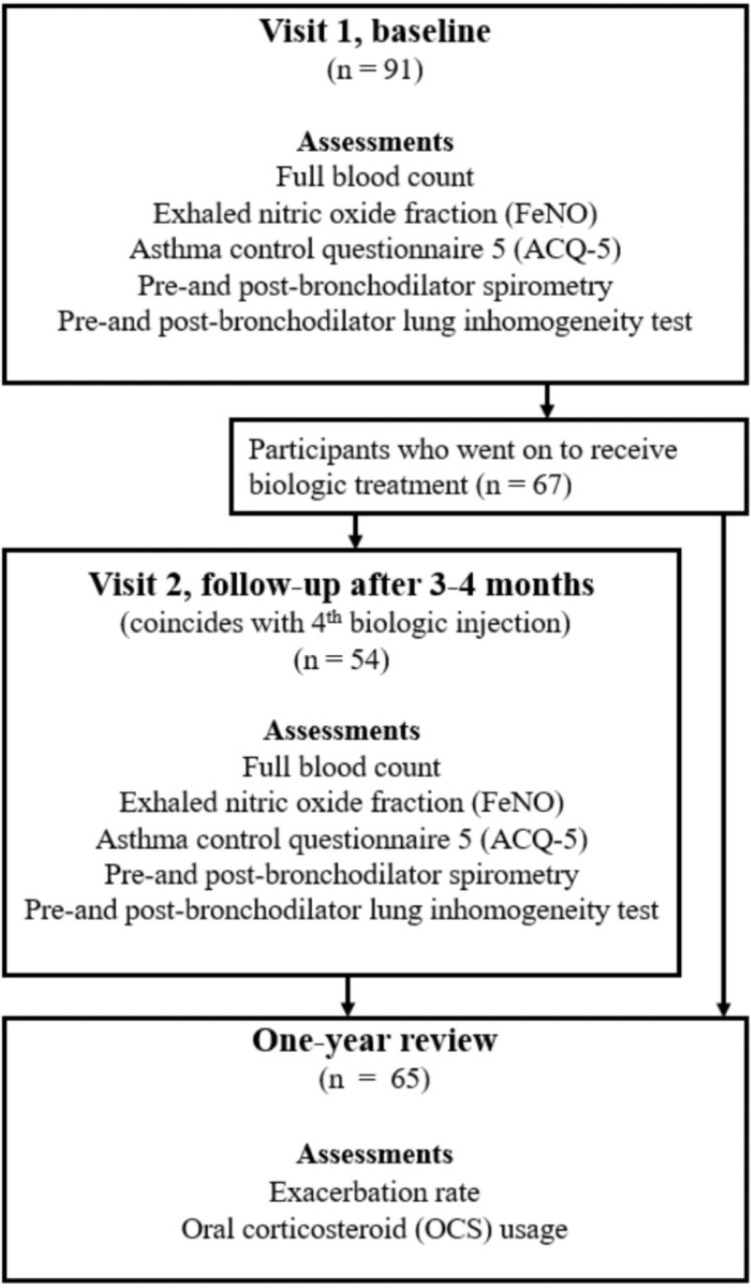
Flow diagram for the observational study of patients within the clinical care pathway for Th2-high asthma.

Patients with severe T2-high asthma were studied at one or two time points alongside their clinical care pathway, as shown in figure 1. All patients had a baseline visit (Visit 1). Those who proceeded to receive biologic therapy [which was decided according to NICE (National Institute of Clinical Excellence) eligibility criteria^14 15^ at the time by their clinicians, irrespective of this study], either anti-IL5 (mepolizumab) or anti-IL5R (benralizumab) monoclonal antibodies, also had a follow-up visit (Visit 2), at either 3 or 4 months after their first biologic injection, respectively, coinciding with their fourth injection. These participants also underwent a clinical review 1 year after starting biologics to evaluate their response to therapy, assessed by reductions in exacerbation frequency, symptoms and oral corticosteroid use (figure 1).

At each visit, participants underwent the following assessments: (1) FeNO measurement; (2) full blood count; (3) Asthma Control Questionnaire (ACQ-5); (4) spirometry: forced expiratory volume in 1 s (FEV_1_), forced vital capacity (FVC) and the ratio of FEV_1_ to FVC (FEV_1_/FVC) and (5) lung inhomogeneity testing using CCP. Spirometry and lung inhomogeneity testing occurred twice at each visit, both before and at least 20 min after bronchodilation (BD) with salbutamol (400 µg) inhaled through a spacer.

### Lung inhomogeneity test using computed cardiopulmonography

Each participant breathed normally through a mouthpiece connected to a novel molecular flow sensor device (MFS)^16^ for 12 min. For the first 7 min they breathed air and for the final 5 min they breathed 100% oxygen for a multiple-breath nitrogen washout. Throughout this period, the MFS used laser absorption spectroscopy and a precise flow metre to record the flux of respired gases (O_2_, N_2_, CO_2_ and water vapour) every 10 ms with very high precision. Using a non-linear fitting technique, the parameters of a cardiopulmonary model were fit to the gas-exchange data, as previously described.^7 8 16^ The fitting process recovered the following participant-specific lung parameters, which constitute novel lung function indices: (1) σlnCl, the SD of the log-normal distribution for (normalised) alveolar compliance. This parameter serves as an indicator of the variability in lung inflation and deflation throughout the breathing cycle across the lung, reflecting the regional variation in lung compliance; (2) σVd, the SD of the normal distribution for (normalised) dead space across lung volume; (3) VD, the volume of the end-inspiratory anatomical dead space and (iv) VA, the alveolar volume at functional residual capacity (FRC).

### Statistical analysis

For comparisons between two groups of values, a Mann-Whitney U test (unpaired data) or a Wilcoxon signed-rank test (paired data) was employed for non-normally distributed data; otherwise, a Student’s or a Welch’s t-test was used, depending on whether the groups had a similar variance or not. For comparisons of frequencies, a χ^2^ test or Fisher’s exact test was used. Correlations between variables were examined using Pearson or Spearman correlation coefficients. To assess the effects of various factors on spirometric and CCP variables, linear mixed-effects (LME) regression was employed to account for repeated observations within an individual (RStudio, nlme package). Several different models were employed according to the particular analysis and took the form:

1.
Y∼BD+BEC+FeNO+ACQ−5+(1|Participant),

where Y, the dependent variable is typically a spirometric (eg, FEV_1_ % predicted) or CCP parameter (eg, σlnCl); fixed effects included the binary fixed effect BD (taking the value 0 for measurements taken pre-BD, and 1 for measurements post-BD) and the continuous variables FeNO, baseline BEC and ACQ-5; and participant was a random factor.

Models assessing the effects of biologics on spirometric or CCP parameters took the form:

1.
$\begin{array}{l} Y∼BD+Visit+(type\;of\;biologic)+\\ \;Visit\times (type\;of\;biologic)+Visit\times BD+(1|Participant), \end{array}$

where additional binary fixed effects included Visit (0 for ‘before biologics-Visit 1’ and 1 for ‘after biologics-Visit 2’), type of biologic (benralizumab or mepolizumab), and the interactive terms Visit by type of biologic and Visit by pre/post-BD.

Non-significant terms were sequentially removed from the model, least significant first. To compare the quality of fit of different models, the Akaike Information Criterion (AIC) was employed.^17^ Statistical significance was assumed at p<0.05.

## Results

### Participants

91 participants with severe T2-high asthma enrolled in the study. Of these, 67 subsequently commenced biologics. The original plan was that all participants would also undertake a CCP assessment on a second visit, 3 or 4 months after starting biologics. However, because of the intervening COVID-19 pandemic, it was only possible for 54 of these second visits to take place. Table 1 lists the physical and phenotypic baseline characteristics of the participants.

**Table 1:** Severe T2-high asthma patient characteristics at baseline

Characteristics	SA-all	SA-biologics	SA-not on biologics
Number of participants	91	67	24
Female (Number, % in brackets)	45 (50)	36 (54)	9 (37.5)
Age (yr)	57±12	59±11	52±16
Height (m)	1.69±0.09	1.68±0.09	1.72±0.07
Weight (kg)	81±17	82±18	77±14
BMI (kg/m^2^)	28.3±6.3	30±6.5	26±5
Exacerbations (/yr)	NA	5±3	NA
OCS maintenance (Number, % of patients in brackets)	25 (27)	25 (37)	0
Blood eosinophil count (x10^9^/L)	0.46±0.40	0.43±0.40	0.57±0.40
FeNO (ppb)	67±62	54±54	103±70
ACQ-5 score	2.3±1.2	2.4±1.3	2.2±1.0

Values are means±SD.SD.

ACQ-5, Asthma Control Questionnaire-5biologics, subset of SA patients who did not go on biologics; BMI, body mass index; FeNO, fractional concentration of exhaled nitric oxide; NA, not applicable as the assessment of baseline exacerbations was done only on patients who received biologics; OCS, oral corticosteroids; SA, severe asthma patients; SA-biologics, subset of SA patients prescribed biologics

### Baseline physiological data for all Th2-high severe asthma patients

Prebronchodilator and postbronchodilator values for spirometry and CCP parameters at baseline are given in table 2.

**Table 2:** Effects of bronchodilation on baseline spirometric and CCP parameters in all patients with severe type-2 high asthma

Variables	Mean	SD	95% CI of the difference(lower to upper)	P value
Spirometry(n=91 patients)				
Pre-BD FEV_1_ (% predicted)	75	20		
Post-BD FEV_1_ (% predicted)	82	19		
Δ FEV_1_ (% predicted)	7.0		5.6 to 8.5	<0.001
Δ FEV_1_ (mL)	203		161 to 244	<0.001
Pre-BD FVC (% predicted)	92	19		
Post-BD FVC (% predicted)	97	17		
Δ FVC (% predicted)	5.1		3.2 to 7.1	<0.001
Δ FVC (mL)	144		80 to 210	<0.001
Pre-BD FEV_1_/FVC	0.65	0.12		
Post-BD FEV_1_/FVC	0.69	0.13		
Δ FEV_1_/FVC	0.04		0.02 to 0.05	<0.001
Computed cardiopulmonography(n=84 pre-BD, n=79 post-BD, n=77 paired data)				
Pre-BD Vd (L)	0.161	0.069		
Post-BD Vd (L)	0.167	0.076		
Δ Vd (L)	0.003		−0.006 to 0.012	
Pre-BD FRC (L)	2.960	0.883		
Post-BD FRC (L)	3.005	1.237		
Δ FRC (L)	−0.032		−0.095 to 0.031	
Pre-BD σVd	0.40	0.11		
Post-BD σVd	0.37	0.12		
Δ σVd	−0.03		−0.05 to −0.01	<0.01
Pre-BD σlnCl	0.83	0.21		
Post-BD σlnCl	0.78	0.27		
Δ σlnCl	−0.08		−0.10 to −0.05	<0.001

BD, bronchodilation; CCPcomputed cardiopulmonography FEV_1_, forced expiratory volume in 1 s; FRC, functional residual capacity; FVC, forced vital capacity; VD, end-inspiratory dead space volume; σlnCl, SD for the natural logarithm for the standardised lung complianceσVd, SD for the standardised dead space

BD significantly improved spirometric variables FEV_1_ % predicted, FVC % predicted and FEV_1_/FVC. For CCP parameters, BD significantly improved (reduced) the inhomogeneity indices σlnCl and σVd (p<0.001, for each) but did not affect anatomical deadspace (VD) or FRC.

**Figure 2:**
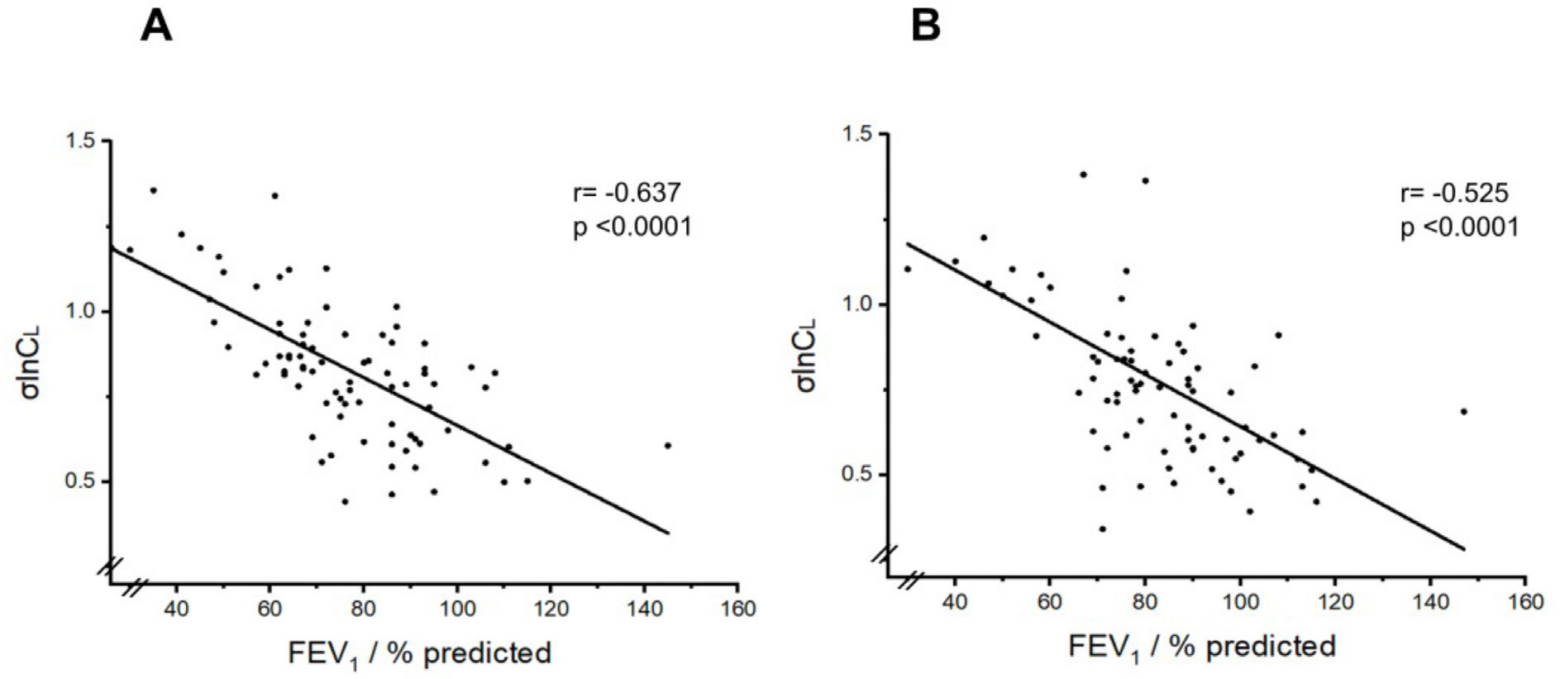
Relationship between σlnCl and FEV_1_ % predicted. (A) Prebronchodilator values. (B) Postbronchodilator values. Pearson correlation coefficients are shown. FEV_1_, forced expiratory volume in 1 s.

The relationship between σlnCl and FEV_1_ % predicted is illustrated in figure 2. There is a significant correlation between the two variables (Pearson correlation coefficient r=−0.64 for pre-BD and r=−0.53 for post-BD values, p<0.0001 for each), confirming that σlnCl is a good marker of progressive lung function impairment in patients with asthma.

We then explored the relationship of these physiological parameters with various baseline disease markers such as BEC, FeNO and ACQ-5 using an LME model (Model 1). As shown in table 3, FEV_1_ % predicted and FVC % predicted were each significantly related to ACQ-5, such that a higher ACQ-5 score was associated with a lower FEV_1_ % predicted or FVC % predicted (p<0.001, respectively), but were not related to BEC. In contrast, σlnCl was significantly related to BEC, such that a higher BEC was associated with a higher σlnCl (p<0.02), but not related to ACQ-5. FeNO had no significant effect on any spirometric or CCP parameters.

**Table 3:** Linear mixed-effects modelling: Effects of selected variables (disease markers) at baseline, on baseline spirometric and CCP inhomogeneity parameters in patients with severe type-2 high asthma

Fixed effects	Est coefficient	95% CI (lower, upper)	dF	P value
Dependent variable: FEV_1_ % pred				
Intercept	90.1	(81.1, 99.1)	82	p<0.001
BD	6.5	(5.1, 7.9)	82	p<0.001
ACQ-5	−4.9	(−8.2, –1.6)	80	p=0.005
BEC (x10^9^/L)	−8.6	(−18.8, 1.6)	80	p=0.102
Dependent variable: FVC % pred				
Intercept	104.9	(97.3, 112.5)	82	p<0.001
BD	5.5	(3.7, 7.3)	82	p<0.001
ACQ5	−5.6	(−8.5, –2.7)	82	p<0.001
Dependent variable: σlnCl				
Intercept	0.78	(0.70, 0.86)	83	p<0.001
BD	−0.08	(−0.10, –0.06)	75	p<0.001
BEC (x10^9^/L)	0.18	(0.04, 0.32)	83	p=0.013
Dependent variable: σVd				
Intercept	0.39	(0.35, 0.43)	83	p<0.001
BD	−0.03	(−0.05, –0.01)	75	p=0.003
BEC (x10^9^/L)	0.04	(−0.02, 0.10)	83	p=0.191

Note, non-significant terms were sequentially removed from the model with the least significant first (only included variables with p- value of <0.2).

ACQ-5, Asthma Control Questionnaire-5; BDbronchodilationBECblood eosinophil countdF, degrees of freedom; Est, estimated; FeNOfractional exhaled nitric oxideFEV_1_, forced expiratory volume in 1 s; FRC, functional residual capacity; FVC, forced vital capacity; σlnCl, SD for the natural logarithm for the standardised lung complianceσVdstandard deviation for the standardised dead space

### Effects of biologic therapy

For the subset of patients (n=67) that received biologics, the effects of BD, and the influence of BEC, FeNO, ACQ-5 and baseline exacerbation frequency on spirometric and CCP parameters at baseline were generally consistent with those in the large parent dataset (online supplemental table 1).

**Figure 3:**
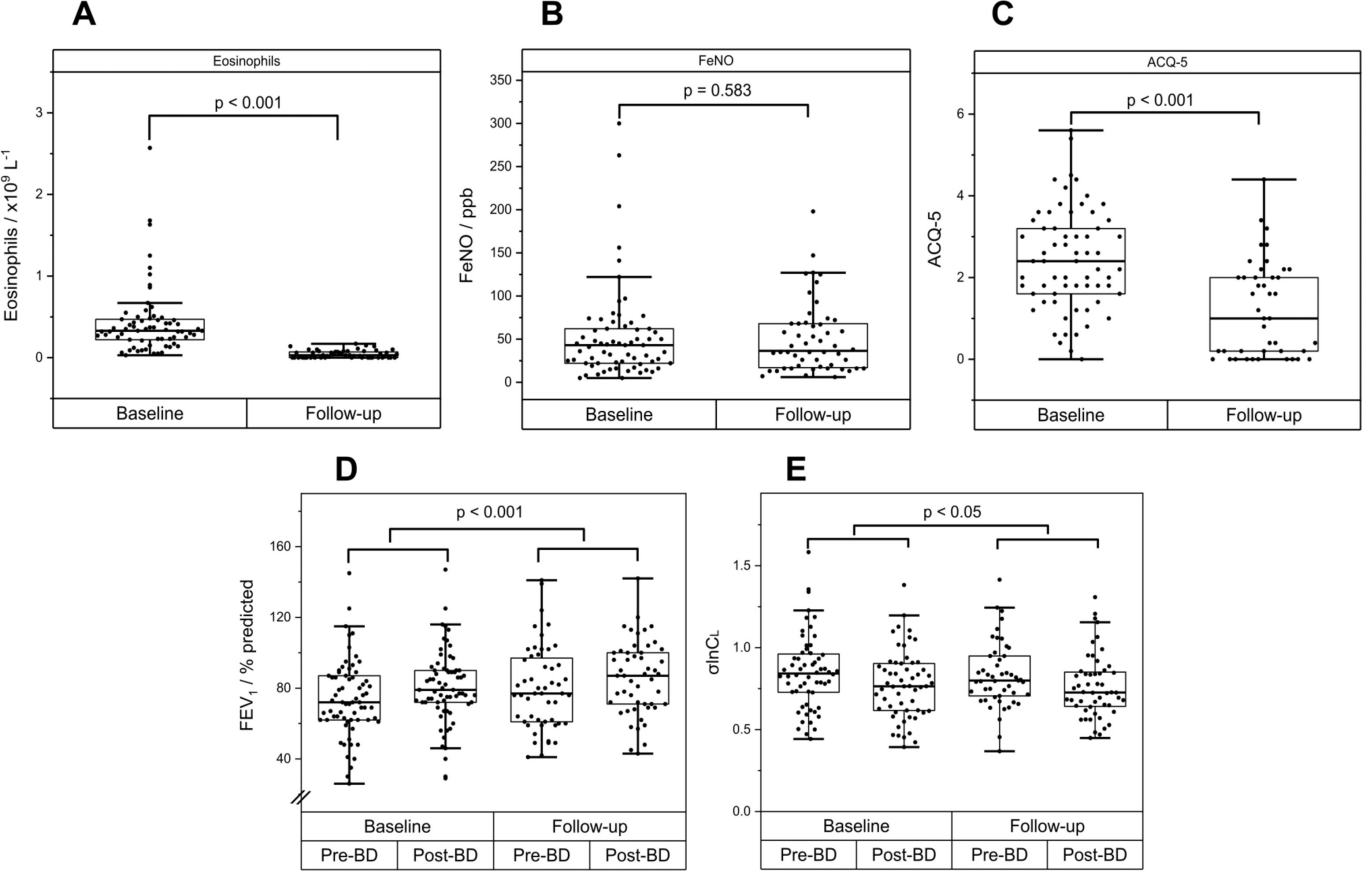
Response to biologics. (A) Blood eosinophils. (B) Fraction of exhaled nitric oxide (FeNO). (C) Asthma Control Questionnaire 5 (ACQ-5). (D) Forced expired volume in 1 s (FEV_1_ % predicted). (E) SD of the natural logarithm of the standardised lung compliance (σlnCl).

The effects of biologics on these variables are illustrated in figure 3A–C. The administration of biologics greatly reduced the BEC (figure 3A, Δ BEC=−0.41×10^9^ cells/L, 95% CI (−0.43×10^9^, –0.28 × 10^9^), p<0.001) as expected, and also significantly reduced ACQ-5 (figure 3C, Δ ACQ-5=−0.86, 95% CI (−1.22, –0.51), p<0.001), but had no effect on FeNO (figure 3B, Δ FeNO of 2 ppb, 95% CI (−8, 12), p=NS).

Figure 3D,E shows the effects of biologics on FEV_1_% predicted and σlnCl. LME Model 2 was used to analyse the effects of biologics on spirometric and CCP inhomogeneity parameters. We found that FEV_1_ % predicted and FVC % predicted were each significantly modified (increased) by biologics (FEV_1_: coefficient for Visit +5.8 % pred, 95% CI (3.5, 8.2), p<0.001, and FVC: coefficient for Visit +4.4 % pred, 95% CI (2.0, 6.8), p<0.001 for the effect of Visit, online supplemental table 2). There was no difference between biologics (mepolizumab or benralizumab) in this effect. From the CCP parameters, only σlnCl was significantly improved (reduced) by biologics (coefficient for Visit −0.04, 95% CI (−0.08 to –0.00), p<0.02, online supplemental table 2), and this again was not influenced by the type of biologic.

**Figure 4:**
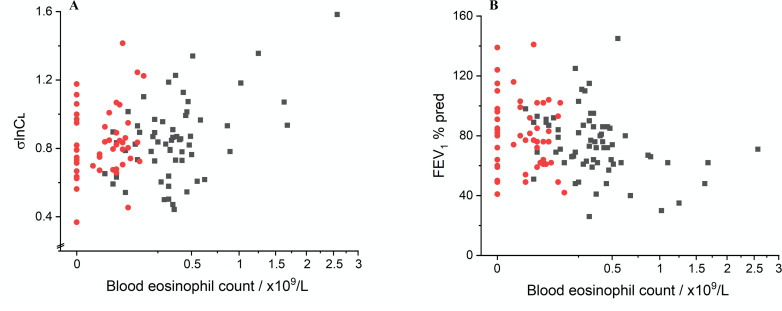
Relationship between (**A**) σlnCl and (**B**) FEV_1_ % predicted with the peripheral blood eosinophil count. Black squares represent baseline (pre-biologic) measurements, and red dots postbiologic measurements. FEV_1_, forced expiratory volume in 1 s.

The change in σlnCl following biologics was modest. Given that biologic therapy had very significant effects on BEC and ACQ-5 (figure 3A,C), it raised the question of whether any effect of biologic therapy on spirometric and CCP parameters related to the magnitude of the biologic effect on BEC and ACQ-5. The relationship of BEC with σlnCl and FEV_1_% pred is illustrated in figure 4. To address this question, BEC and ACQ-5 (as well as FeNO) were incorporated into the LME model to assess their association with physiological parameters measured before and after biologics. Results are detailed in online supplemental table 3. This analysis revealed a large significant effect of BEC on the magnitude of σlnCl (coefficient +0.19, 95% CI (0.11, 0.27), p<0.001) and a significant effect on σVd (coefficient +0.05, 95% CI (0.01, 0.09), p<0.01). For spirometry, there was a significant relationship of both ACQ-5 and BEC with FEV_1_ % predicted (ACQ5: coefficient −3.5 % pred, 95% CI (−5.1 to –1.9), p<0.001; BEC: coefficient −10.8 % pred, 95% CI (−16.1, 5.5), p<0.001) and FVC % predicted (ACQ5: coefficient −2.5 % pred, 95% CI (−4.3 to –0.7), p<0.005; BEC: coefficient −8.2 % pred, 95% CI (−13.7, –2.7), p<0.005). These results suggest that any effect induced by biologics on inhomogeneity parameters significantly (and solely) depends on the BEC response, whereas for spirometric parameters, it is related to the biologics effect on both BEC and ACQ5, that is, an improved FEV_1_ % predicted associated with a reduced ACQ5 and a reduced BEC.

**Figure 5:**
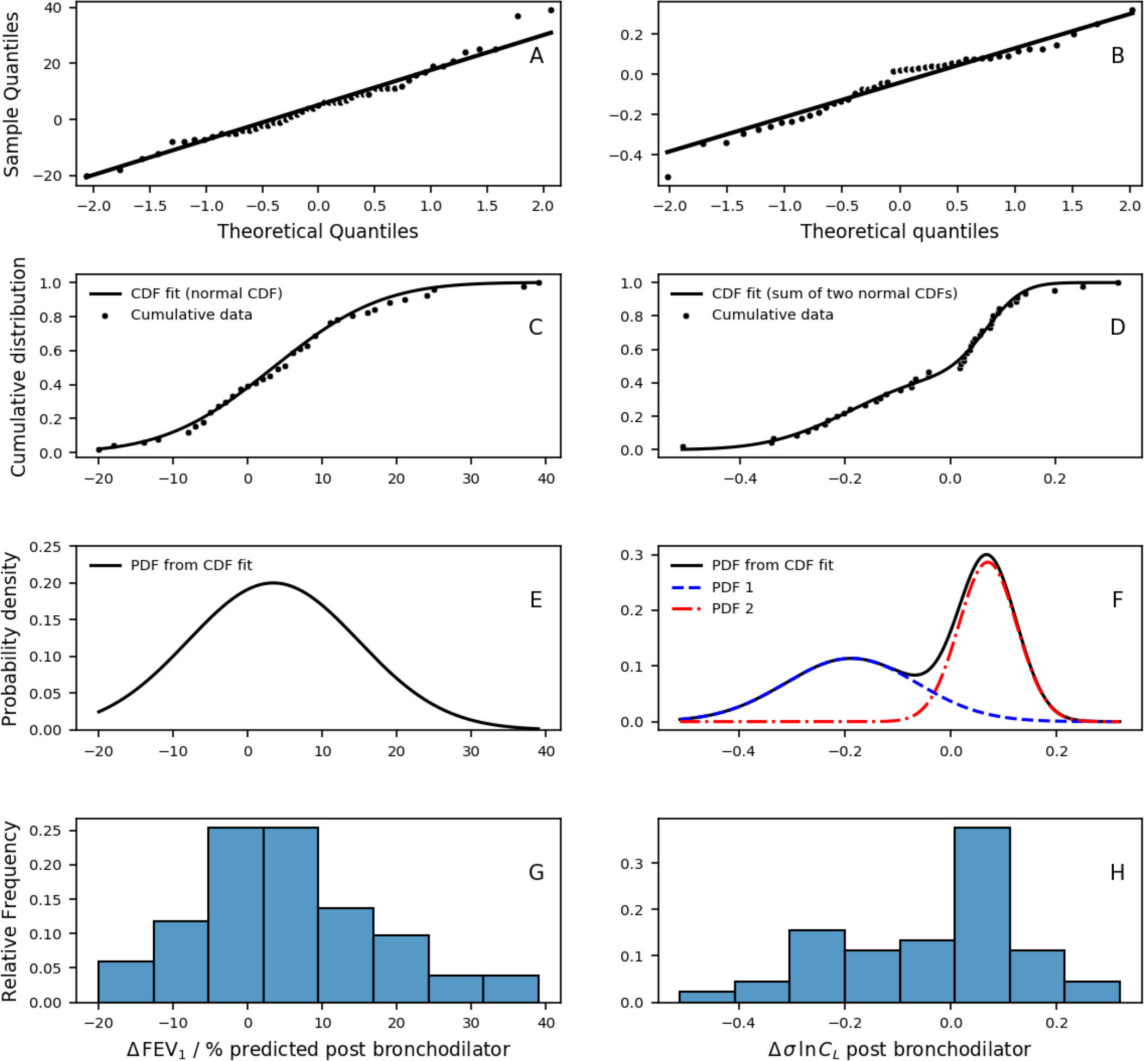
Effect of biologics on postbronchodilator (post-BD) values for values for FEV_1_ % predicted (left) and σlnCl (right). Δ FEV_1_ % predicted and Δ σlnCl represent change in post-BD FEV_1_ % predicted and σlnCl following biologics, respectively. (A,B) Q-Q plots. (C,D) Cumulative distributions, data and model fit. (E,F) Probability density functions, illustrating underlying bimodal fit for σlnCl (**D**). (G,H) Sample histograms. FEV_1_, forced expiratory volume in 1 s.

**Figure 6:**
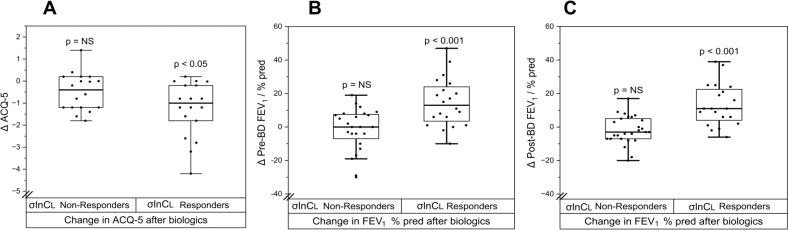
Change in symptoms and lung function after starting biologic therapy between ‘σlnCl Non-Responders’ and ‘σlnCl Responders’. (**A**) Change (Δ) in ACQ-5 postbiologics. (**B**) Change (Δ) in prebronchodilator (BD) FEV_1_ % predicted postbiologics; absolute change in FEV_1_ was −20 mL versus +330 mL in σlnCl Non-Responders versus σlnCl Responders, and (**C**) Change (Δ) in post-BD FEV_1_ % predicted postbiologics; absolute change in FEV_1_ was −40 mL versus +350 mL in σlnCl Non-Responders versus σlnCl Responders. There was a significant reduction in ACQ-5 and a significant increase in FEV_1_ % predicted (both for pre- and post-BD values) in σlnCl Responders, but not σlnCl Non-Responders. FEV_1_, forced expiratory volume in 1 s.

Further inspection of the data suggested that there may be two distinct groups of patients for the change in post-BD σlnCl with biologics (figure 5), but not for the change in FEV_1_ % predicted. This was confirmed by demonstrating that a bimodal distribution (weighted sum of two normal distributions) fitted the σlnCl response data significantly better than a unimodal normal distribution (AIC −216 vs −144, bimodal vs unimodal, p<0.05), enabling the patients to be classified into two groups: ‘σlnCl Non-Responders’ and ‘σlnCl Responders’. Importantly, we found that ‘σlnCl Responders’ demonstrated significant improvements in symptoms (ACQ-5) and FEV_1_ % predicted (both pre- and post-BD) following biologics, whereas ‘σlnCl Non-Responders’ did not, as shown in figure 6. Other comparisons between the two groups are shown in online supplemental table 4.

We then proceeded to examine whether we could identify any predictors of the clinical response to biologics assessed after 1 year. When we classified our study participants into ‘Clinical Responders’—that is, ≥50% reduction in their annual exacerbation rate and ≥50% reduction in their use of maintenance oral corticosteroids—and ‘Clinical Non-Responders’, we found that all but one were ‘Clinical Responders’. This meant that this classification was not particularly useful in this real-world patient cohort for seeking to identify predictors of clinical response. As such, we sought to identify patients who achieved clinical remission (CR) after 1 year of biologics therapy versus those who did not. Patients were classified as achieving CR if they met all of the following conditions: zero exacerbations, zero maintenance oral corticosteroid therapy for their asthma, ACQ-5 <1.5, and post-BD FEV_1_% predicted ≥80% or change in pre-BD FEV_1_ of >100 ml.^18^ For the FEV_1_ criterion, we used data from Visit 2 (post-biologics) as not all patients had spirometry at 1 year (due to the COVID-19 pandemic, which meant their 1-year clinical assessment was often a virtual one). We tested whether the proportion of patients in CR differed between the ‘σlnCl Responders’ versus ‘σlnCl Non-Responders’. We found only 3 out of 24 participants achieved CR in the ‘σlnCl Non-Responders’ (12.5%) versus 8 out of 21 achieving CR in the ‘σlnCL Responders’ group (40%), p<0.05.

## Discussion

This is the first study to assess the novel CCP-derived indices of lung inhomogeneity in patients with severe T2-high asthma and the effects of therapeutics (biologics and bronchodilators) on these indices.

σlnCl values in this severe asthma patient group appear to be highly abnormal: if we compare these σlnCl values with those obtained in other studies for which there are healthy control participants—for example, Smith *et al*^12^ in which healthy participants had pre-BD σlnCl of 0.45±0.09 (mean±SD) and asthma patients 0.76±0.24—it is clear that the values in these severe asthma patients normally exceed the upper bound of those for all healthy participants. BD with salbutamol significantly improved lung inhomogeneity parameters relating to ventilation (σlnCl) and dead space unevenness (σVd) in patients with severe type-2 high asthma.

σlnCl, a novel measure of ventilation inhomogeneity, was significantly associated with the level of systemic eosinophilic inflammation, measured by the peripheral BEC, in this patient cohort when studied before commencing biologics. In contrast, FEV_1_% predicted appeared to have a stronger relationship with symptoms (ACQ-5 score) than with BEC. We also showed that while σlnCl significantly correlated with FEV_1_ % predicted, there was significant variation around the regression line, signifying that it is not a direct proxy for it (figure 2). This likely relates to the fact that markers of ventilation inhomogeneity typically capture disease in the peripheral lung zone (small airways), while spirometry (FEV_1_% predicted) is predominantly influenced by large airway dysfunction. As such, taken together, these results suggest that higher levels of systemic eosinophilic inflammation are associated with a higher disease burden in the peripheral lung, reflected in higher σlnCl values, while the disease or airflow limitation (in many cases fixed) captured by FEV_1_ is less dependent on the degree of eosinophilic inflammation and more closely related to symptoms.

Small-airways disease or dysfunction (SAD) is highly prevalent in asthma.^19^ Previous studies found that the prevalence of SAD, measured by different physiological tests such as FEF_25-75_, impulse oscillometry and conventional multiple breath washout (MBW) indices [Lung Clearance Index (LCI), S_acin_ and S_cond_], increased progressively with GINA asthma stage.^20^ Furthermore, SAD correlated with clinically important outcomes such as asthma control and exacerbations.^21 22^ These studies included a wide population of asthma patients with varying disease severity (mild to severe) and were not confined to a specific inflammatory phenotype. Our study included only patients with severe (≥GINA step 4) type-2 high asthma. Nonetheless, even within this stringently defined patient group, our results are consistent with these studies: patients with the highest frequency of exacerbations (>6 per year, in 17 patients), signifying high disease burden, had significantly higher σlnCl values at 1.00±0.24, mean±SD (pre-BD), compared with those with fewer exacerbations at 0.79±0.18 (p<0.001).

Anti-IL5 and anti-IL5R biologic therapies specifically target eosinophilic inflammation, suppressing or depleting eosinophilic activation, proliferation and migration, causing a significant reduction in BEC.^23^,^25^ In this study, we chose to review changes in lung inhomogeneity and lung function at patients’ fourth biological injection, as this coincided with their clinical review, but also assess whether there is an ‘early treatment response signal’ in measures of disease activity within the lung. We found that after 3–4 months, there was a statistically significant and clinically important improvement in symptoms (mean ACQ5 change of −0.86, p<0.001, figure 3). We also found an improvement in lung function (FEV_1_% predicted) and in σlnCl, suggesting that targeting eosinophilic inflammation does affect disease activity within the lung and has a functional impact early on their commencement. Importantly, our further analysis showed that, for σlnCl, any effect of biologics was mainly, and very significantly, related to the degree of eosinophilic inflammation as reflected by BEC (figure 4). This reinforces the notion that reducing systemic eosinophilic inflammation is associated with effects on disease burden in the peripheral lung regions.

Our results are consistent with Farah *et al*^26^ who demonstrated in a prospective cohort of 20 adults a significant improvement in spirometry and the MBW indices LCI and S_acin_ after 4 weeks of mepolizumab, which was sustained at 26 weeks. They suggested that targeting eosinophilic inflammation systemically via biologics might allow access to the biological effect of the small airways, unlike inhaled therapies. They further suggested that changes in the peripheral airway compartment are also contributing to the improvements seen in FEV_1_. In their study, following mepolizumab changes in MBW indices, but not changes in spirometry, correlated with changes in symptoms (ACQ-5). This contrasts our results, in which for FEV_1_% predicted, the effect of biologics was associated with both the effect on eosinophil inflammation and the degree of symptom (ACQ-5) improvement, unlike effects on σlnCl which were mainly related to the blood eosinophilic response. Other studies examining changes in small-airway function at a much later time point (>6 months) following anti-IL5/IL5R biologics reported mixed results: MUSCA, a randomised trial of mepolizumab versus placebo in severe eosinophilic asthma, demonstrated improvements in FEF_25-75_ after 24 weeks.^27^ Similar improvements in FEF_25-75_ were seen in retrospective observational studies with mepolizumab^28 29^ and benralizumab.^30 31^ When small-airways function was assessed with impulse oscillometry, the results were less consistent.^32^,^34^ The highly variable results from these and other SAD studies^19^ highlight the lack of consensus on which small-airways function measures should be used and that perhaps different measures assess different aspects of pathophysiology. More work is needed in this area.

The most important observation in our study is the ability of our test to separate participants into Responders and Non-Responders based on their change in σlnCl. Such dichotomisation of response was not possible with spirometry (FEV_1_). This dichotomisation revealed that when biologics were able to cause a substantial change in disease activity within the lung (measured by σlnCl), those patients also benefitted from an improvement in symptoms and importantly overall lung function as assessed by spirometry (figure 6). From this dichotomisation, it appears that fully optimised standard therapy (eg, through GINA steps) still leaves a subset of patients with ongoing inflammation within their lungs inadequately treated (indeed σlnCl Responders had worse lung function at baseline), which can be modified with biologics; and that changes in disease activity *within* the lung induced by biologics are to a great extent independent of whether these biologics reduce the frequency of T2-driven exacerbations, which by-and-large they always do (in virtually all of our patients) through suppressing blood eosinophils. This observation raised the possibility that CCP assessment and stratification of patients as Responders versus Non-Responders for σlnCl could act as an ‘early response signal’ that related to or predicted the longer-term clinical response to biologic therapy. Indeed, our results suggested that those with an early improvement in σlnCl were more likely to respond to biologic therapy with CR at 1 year, compared with those with no change in σlnCl, but larger studies are required to test this observation more robustly.

There are several limitations in our study: first, it is an observational study conducted in a real-life patient cohort alongside their clinical care pathway (as opposed to a randomised-controlled trial), without a control group; this leads to more heterogeneity in the participants and also the data obtained. Furthermore, the intervening COVID-19 pandemic limited the number of paired visits for some patients.

Furthermore, due to the eligibility criteria which depend both on biomarkers and the presence of multiple exacerbations,^14 15^ patients commencing biologics in the UK, particularly during the period of our study, have generally been those with severe chronic disease associated with significant airway remodelling and permanent lung damage. Markers of ventilation inhomogeneity or small-airway disease, including the novel CCP indices, are likely to be more useful in disease which are associated with less damage as they could identify the presence of significant small-airway disease burden despite preserved spirometry. Future clinical trials are likely to assess the efficacy of earlier introduction of biologics in less severe patients with active disease before significant lung damage has occurred.^35^ The predictive power of novel small-airway indices as early treatment-response signals or their use as endpoints should be evaluated in such research trials.

In summary, our main finding is that the novel measure of ventilation inhomogeneity, σlnCl, produced by CCP, is most closely related to systemic eosinophilic inflammation (both for baseline values and for the changes induced by biologics) in patients with severe type-2 high asthma; and that its early response to biologics identifies patients more likely to benefit with improvements in symptoms, lung function and potentially long-term remission.

## supplementary material

10.1136/bmjresp-2024-002721 online supplemental file 1
