# Supplementary material for: Effects of biologic therapy on novel indices of lung inhomogeneity in patients with severe type-2 high asthma
Source: BMJ Open Respir Res. 2025 Feb 8;12(1):e002721. doi: 10.1136/bmjresp-2024-002721 (PMC11808925; doi:10.1136/bmjresp-2024-002721)
Supplement: online supplemental file 1 [file bmjresp-12-1-s001.docx]

**SUPPLEMENTAL MATERIAL**

**Supplementary Tables.**

**Supplementary Table 1.** Linear, mixed-effects modelling: Effects of selected variables (disease markers) at baseline, on baseline spirometric and CCP parameters in the sub-cohort of patients who subsequently received biologics.

| ***Dependent Variable: FEV_1_ % pred*** | | | | |
| --- | --- | --- | --- | --- |
| **Fixed Effects** | **Est Coefficient** | **95% CI**  **[Lower, Upper]** | **dF** | **p-value** |
| Intercept | 89.8 | [79.3, 100.4] | 64 | p<0.001 |
| BD | 6.2 | [4.6, 7.8] | 64 | p<0.001 |
| ACQ5 | -4.9 | [-8.8, -1.0] | 62 | p=0.019 |
| BEC (x10^9^ L^-1^) | -9.8 | [-21.6, 2.0] | 62 | p=0.106 |
| ***Dependent Variable: FVC % pred*** | | | | |
| **Fixed Effects** | **Est Coefficient** | **95% CI**  **[Lower, Upper]** | **dF** | **p-value** |
| Intercept | 108.0 | [98.8, 117.2] | 63 | p<0.001 |
| BD | 5.0 | [2.8, 7.2] | 63 | p<0.001 |
| ACQ5 | -7.0 | [-10.3, -3.7] | 61 | p<0.001 |
| FENO | 0.1 | [0.0-0.2] | 61 | p=0.056 |
| BEC (x10^9^ L^-1^) | -7.5 | [-17.3, 2.3] | 61 | p=0.143 |
| ***Dependent Variable: σlnC_L_*** | | | | |
| **Fixed Effects** | **Est Coefficient** | **95% CI**  **[Lower, Upper]** | **dF** | **p-value** |
| Intercept | 0.76 | [0.68, 0.84] | 60 | p<0.001 |
| BD | -0.08 | [-0.12, -0.04] | 54 | p<0.001 |
| BEC | 0.21 | [0.09, 0.33] | 60 | p<0.001 |
| ***Dependent Variable: σVD*** | | | | |
| **Fixed Effects** | **Est Coefficient** | **95% CI**  **[Lower, Upper]** | **dF** | **p-value** |
| Intercept | 0.40 | [0.36, 0.44] | 60 | p<0.001 |
| BD | -0.03 | [-0.05, -0.01] | 54 | p=0.022 |
| BEC (x10^9^ L^-1^) | 0.04 | [-0.02, 0.10] | 60 | p=0.182 |

CI: Confidence Interval; Df, degrees of freedom; Est, estimated; ACQ-5, asthma control questionnaire-5, FeNO, fractional exhaled nitric oxide, BEC, blood eosinophil count, BD, bronchodilation; FEV_1_, forced expiratory volume in 1s; FVC, forced vital capacity; standard deviation for the standardised deadspace; FRC, functional residual capacity; σlnCL, standard deviation for the natural logarithm for the standardised lung compliance. Note non-significant terms were sequentially removed from the model with the least significant first (only include variables with p-value of <0.2).

***Supplementary Table 2.*** *Linear, mixed-effects modelling: Effects of biologic therapy on spirometric and computed cardiopulmonography parameters in patients with severe Type-2 high asthma.*

| ***Dependent Variable: FEV_1_ % pred*** | | | | |
| --- | --- | --- | --- | --- |
| **Fixed Effects** | **Est Value** | **95% CI**  **[Lower, Upper]** | **dF** | **p-value** |
| Intercept | 74.2 | [69.3, 79.1] | 168 | p<0.001 |
| BD | 6.0 | [3.8, 8.2] | 168 | p<0.001 |
| Visit | 5.8 | [3.5, 8.2] | 168 | p<0.001 |
| ***Dependent Variable: FVC % pred*** | | | | |
| **Fixed Effects** | **Est Value** | **95% CI**  **[Lower, Upper]** | **dF** | **p-value** |
| Intercept | 93.1 | [88.6, 97.6] | 167 | p<0.001 |
| BD | 3.4 | [1.2, 5.6] | 167 | p=0.004 |
| Visit | 4.4 | [2.0, 6.8] | 167 | p<0.001 |
| ***Dependent Variable: σlnC_L_*** | | | | |
| **Fixed Effects** | **Est Value** | **95% CI**  **[Lower, Upper]** | **dF** | **p-value** |
| Intercept | 0.85 | [0.79, 0.91] | 151 | p<0.001 |
| BD | -0.07 | [-0.11, -0.03] | 151 | p<0.001 |
| Visit | -0.04 | [-0.08, -0.00] | 151 | p=0.019 |
| ***Dependent Variable: σVD*** | | | | |
| **Fixed Effects** | **Est Value** | **95% CI**  **[Lower, Upper]** | **dF** | **p-value** |
| Intercept | 0.41 | [0.39, 0.43] | 151 | p<0.001 |
| BD | -0.03 | [-0.05, -0.01] | 151 | p=0.021 |
| Visit | -0.02 | [-0.04, -0.00] | 151 | p=0.081 |

Note: visit*type of biologic was non-significant and hence removed from the model, as was type of biologic.

***Supplementary Table 3.*** *Linear, mixed-effects modelling: Exploring the effects of changes induced by biologic therapy on blood eosinophils, the fraction of exhaled nitric oxide and ACQ-5 symptom score on spirometric and computed cardiopulmonography parameters in the subgroup of patients who had a follow-up visit at 3-4 months after starting biologic therapy.*

| ***Dependent Variable: FEV_1_ % pred*** | | | | |
| --- | --- | --- | --- | --- |
| **Fixed Effects** | **Est Value** | **95% CI**  **[Lower, Upper]** | **dF** | **p-value** |
| Intercept | 86.9 | [81.4, 92.4] | 144 | p<0.001 |
| BD | 5.9 | [3.9, 7.9] | 144 | p<0.001 |
| ACQ5 | -3.5 | [-5.1, -1.9] | 144 | p<0.001 |
| BEC (x10^9^ L^-1^) | -10.8 | [-16.1, -5.5] | 144 | p<0.001 |
| ***Dependent Variable: FVC % pred*** | | | | |
| **Fixed Effects** | **Est Value** | **95% CI**  **[Lower, Upper]** | **dF** | **p-value** |
| Intercept | 102.1 | [97.0, 1007.2] | 143 | p<0.001 |
| BD | 3.7 | [1.5, 5.9] | 143 | p=0.001 |
| ACQ5 | -2.5 | [-4.3, -0.7] | 143 | p=0.004 |
| BEC (x10^9^ L^-1^) | -8.2 | [-13.7, -2.7] | 143 | p=0.004 |
| ***Dependent Variable: σlnC_L_*** | | | | |
| **Fixed Effects** | **Est Value** | **95% CI**  **[Lower, Upper]** | **dF** | **p-value** |
| Intercept | 0.75 | [0.69, 0.81] | 128 | p<0.001 |
| BD | -0.07 | [-0.09, -0.05] | 128 | p<0.001 |
| BEC (x10^9^ L^-1^) | 0.19 | [0.11, 0.27] | 128 | p<0.001 |
| ACQ5 | 0.02 | [0.00, 0.04] | 128 | p=0.178 |
| ***Dependent Variable: σVD*** | | | | |
| **Fixed Effects** | **Est Value** | **95% CI**  **[Lower, Upper]** | **dF** | **p-value** |
| Intercept | 0.39 | [0.37, 0.41] | 149 | p<0.001 |
| BD | -0.02 | [-0.04, 0.00] | 149 | p=0.025 |
| BEC (x10^9^ L^-1^) | 0.05 | [0.01, 0.09] | 149 | p=0.008 |

CI: Confidence Interval; Df, degrees of freedom; Est, estimated; ACQ-5, asthma control questionnaire-5, FeNO, fractional exhaled nitric oxide, BEC, blood eosinophil count, BD, bronchodilation; FEV_1_, forced expiratory volume in 1s; FVC, forced vital capacity; standard deviation for the standardised deadspace; FRC, functional residual capacity; σlnCL, standard deviation for the natural logarithm for the standardised lung compliance. Note non-significant terms were sequentially removed from the model with the least significant first (only included variables with p-value of <0.2 here).

***Supplementary Table 4.*** *Comparison of characteristics between the patients that did and did not show a response in post-bronchodilator σlnCL to therapy with biologics at their 4^th^ injection.*

| **Characteristics** | **σlnCL non-responders** | | | **σlnCL responders** | |
| --- | --- | --- | --- | --- | --- |
| Number of participants (% participants) | 24 (53) | | | 21 (47) | |
| OCS (% of patients on maintenance) | 11 (46) | | | 8 (38) | |
| Baseline Exacerbation Frequency/year | 4.8±2.4 | | | 5±3 | |
| Exacerbation Frequency after 1 year /year | 0.46 ± 0.72 | | | 0.29 ± 0.85 | |
| Baseline Blood Eosinophil Count / x10^9^ L^-1^ | | | 0.35±0.34 | 0.52±0.55 | |
| Follow-up Blood Eosinophil Count / x10^9^ L^-1^ | | 0.05±0.05 | | 0.04±0.04 | |
| Δ Blood Eosinophil Count / x10^9^ L^-1^ | -0.31±0.32 | | | -0.48±0.54 | |
| Baseline FeNO / ppb | 46±41 | | | 41±28 |  |
| Follow-up FeNO / ppb | 49±35 | | | 51±44 |  |
| Δ FeNO / ppb | 3±27 | | | 9±36 |  |
| Baseline ACQ-5 score | 2.1±1.2 | | | 2.5 ±1.3 |  |
| Follow up ACQ-5 score | 1.4±1.1 | | | 0.8±0.9 |  |
| Δ ACQ-5 score | -0.5±0.9 | | | -1.3±1.3^*^ |  |
| Baseline pre-BD FEV_1_/ % predicted | 83±24 | | | 68±20^*^ | |
| Follow-up pre-BD FEV_1_ % predicted | 81±25 | | | 82±23 | |
| Δ FEV_1_ / % predicted | -2±13 | | | 15±15^***^ | |
| Baseline pre-BD FVC / % predicted | 100±19 | | | 89±19 | |
| Follow-up pre-BD FVC / % predicted | 101±24 | | | 102±15 | |
| Δ FVC / % predicted | 1.3±13 | | | 13±15^*^ | |
| Baseline pre-BD FEV_1_ /FVC | 0.66±0.11 | | | 0.59±0.11^*^ | |
| Follow-up pre-BD FEV_1_ /FVC | 0.65±0.13 | | | 0.62±0.11 | |
| Baseline pre-BD VD | 0.144±0.044 | | | 0.185±0.100 | |
| Follow-up pre-BDVD | 0.153±0.051 | | | 0.148±0.055 | |
| Δ VD | 0.01±0.03 | | | -0.05±0.07 | |
| Baseline pre-BD σVD | 0.38±0.10 | | | 0.48±0.11^***^ | |
| Follow-up pre-BD σVD | 0.42±0.11 | | | 0.40±0.12 | |
| Δ σVD | 0.03±0.12 | | | -0.09±0.13^*^ | |
| Baseline pre-BD FRC | 2.9±0.70 | | | 3.1±0.86 | |
| Follow up pre-BD FRC | 3.0±0.65 | | | 3.2±0.9 | |
| Δ FRC | 0.08±0.22 | | | -0.07±0.57 | |
| Baseline pre-BD σlnCL | 0.74±0.16 | | | 0.98 ±0.24^***^ | |
| Follow-up pre-BD σlnCL | 0.84±0.21 | | | 0.81±0.20 | |

σlnCL, standard deviation for the natural logarithm for the standardised lung compliance; FEV_1_, forced expiratory volume in 1s; FVC, forced vital capacity; VD, deadspace volume (end inspiratory); σVD, standard deviation for the standardised deadspace; FRC, functional residual capacity; σlnCL, standard deviation for the natural logarithm for the standardised lung compliance. Δ, change in parameter value between baseline and follow up visits. Statistical comparisons are for the σlnCL-Non-Responders with the σlnCL-Responders. Values are means ± SD, *p<0.05, **p<0.01, *** p<0.001.
